# Drug-Induced Liver Injury Caused by Adalimumab: A Case Report and Review of the Bibliography

**DOI:** 10.1155/2013/406901

**Published:** 2013-05-14

**Authors:** Bernardo Frider, Andres Bruno, Marcelo Ponte, Marcelo Amante

**Affiliations:** ^1^Department of Medicine, Hepatology, Hospital General de Agudos Dr. Cosme Argerich, Pi y Margall 750, C1155AHD Buenos Aires, Argentina; ^2^Division of Internal Medicine, Hospital General de Agudos Dr. Cosme Argerich, Pi y Margall 750, C1155AHD Buenos Aires, Argentina; ^3^Division of Pathology, Hospital General de Agudos Dr. Cosme Argerich, Pi y Margall 750, C1155AHD Buenos Aires, Argentina

## Abstract

The most serious adverse drug reaction of adalimumab (ADR) is tuberculosis reactivation. We describe a case of a 35-year-old man, with rheumatoid arthritis (RA) and hepatitis C virus genotype 1a with a liver biopsy in 2001 with a METAVIR score pattern A1 F0; he received interferon alpha 2b for six months, but treatment was suspended because of reactivation of RA. Liver function tests after treatment were similar to previous ones showing a minimal cholestatic pattern. In 2008, methotrexate was prescribed, but the drug was withdrawn at the third month because of the appearance of pruritus and Ggt rise. Viral load at that moment was 9300000 UI/mL, log 6,9. The liver biopsy showed a Metavir Score A2 F1. Adalimumab was started in 2010, and at the third month of treatment, Ggt showed a rise of 23 times normal value (NV), alkaline phosphatase 2,5 times NV with AST and ALT with no change. A new liver biopsy showed portal inflammation with eosinophils and a METAVIR A1 F2. We think that adalimumab appears to be responsible for the liver injury, because of temporal relationship, liver biopsy findings, other clinical conditions being discarded, and the improvement of clinical symptoms and biochemical abnormalities when adalimumab was suspended.

## 1. Introduction

Adalimumab (Humira NR) is a fully humanized monoclonal antibody whose biologic target is tumor necrosis factor alpha (TNF-alpha). This pharmacodynamic mechanism diminishes inflammatory cytokines cascade. The label indications are nowadays rheumatoid arthritis, psoriatic arthritis, ankylosing spondylitis, psoriasis, and Crohn's disease. Clinical studies have demonstrated that the most frequent adverse drug reactions (ADRs) to this drug are the injection site reactions that occur in more than 10% of treated patients, and the most serious ADR is tuberculosis reactivation. Other serious systemic infections, which occur very rarely, are drug-induced systemic lupus erythematosus, lymphoma or demyelinating disease [1–4]. These same studies demonstrate that hepatotoxicity is a rare condition that occurs in less than 5% of treated patients, and asymptomatic elevation of liver enzymes is the most common manifestation of liver toxicity. 

## 2. Case Report

A 35-year-old male patient with a medical history of chronic hepatitis c virus (genotype 1a) since 1999 and rheumatoid arthritis (RA) treated with hydroxychloroquine was examined in the hepatology unit of our Hospital. He showed a liver biopsy done in 2001 with an A1 F0 Metavir score [5] and was treated with interferon alfa 2b (IFN), but this treatment was suspended at the sixth month because of reactivation of RA symptoms. Liver function tests after treatment with IFN were similar to the previous ones, showing a minimal cholestatic pattern: alanine amino transferases (ALT) slightly elevated (×1.5 times the upper normal limit), alkaline phosphatase (AP) 1.6 times the upper normal limit, and gamma-glutamyl-traspeptidase (GgT) 9 times the upper normal limit. The patient did not receive any treatment for his HCV and only received chloroquine (400 mg/d) for RA. 

**Figure 1:**
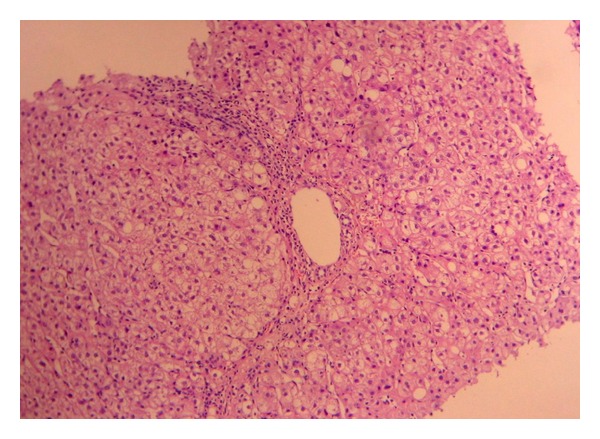
Histology: portal tract with mild lymphocytic inflammatory infiltrate with eosinophils and focal interface hepatitis (HE, 40x).

**Figure 2:**
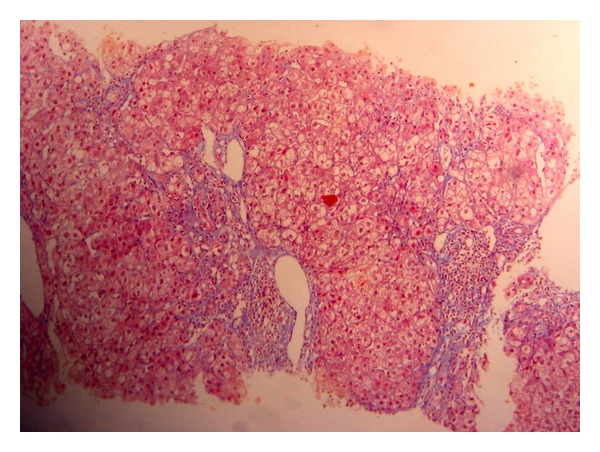
Histology: fibrous portal expansion with short septa. (TRI 40x).

In 2008 methotrexate (MTX) was prescribed, because of RA reactivation and a rise of GgT, and pruritus appeared, and the drug was withdrawn at the third month of onset. Viral load was 9,300,000 UI/mL, (log)6.9. In 2009 the viral load was 4,070,000 UI/mL, log(6.6) and a liver biopsy showed an A2 F1 Metavir score [5]. Adalimumab was started in April 2010, and at the third month liver function tests showed a rise of GgT (23 times the upper normal limit), AP (2.5 times the upper limit) and AST, ALT with no change (1.5 nv), and pruritus. A liver biopsy was then performed with an A1 F2 pattern, portal dystrophy, portal inflammation with eosinophils, lobular hepatitis, and microabscesses (Figures 1 and 2). This pattern was highly suggestive of drug-induced liver injury. Viral load showed a discrete elevation to 8,400,000 UI/mL. Antiliver kidney microsomal (anti-LKM) antibodies, antinuclear antibodies (ANAs), antimitochondrial antibodies (AMAs), and anti-smooth muscle antibodies (ASMAs) were negative. Adalimumab was stopped and liver function tests improved but remained altered (slightly elevated) six months later (AP × 1,5 nv, Ggt × 3 nv). The final diagnosis was adalimumab-induced liver injury with a cholestatic pattern. 

## 3. Discussion

We report a case of adalimumab-induced cholestasis (pruritus, increase of cholestatic enzymes with elevated AST and ALT) in a patient with RA and chronic hepatitis C infection. Drug-induced liver injury (DILI) is one of the most common ADRs and the main cause of drugs withdrawal. In the last few years, several drugs have had to be withdrawn from the market because they were linked to severe liver toxicity. More than 50% of all acute liver failures and near 10% of all acute hepatitis cases are due to drugs nowadays. There are two main types of liver toxicity: the one caused by an immune response triggered by the drug or the one produced by direct toxicity of the drug. The first one is generally dose independent, and it is impossible to recognize who will suffer from this ADR before the drug was administered. The cholestatic pattern is the most frequent in this type of toxicity, and the withdrawal of the drug does not guarantee the solution to the problem, as sometimes the DILI persists long after the drug withdrawal, and in rare cases chronic liver injury may occur owing to a self-perpetuating injury (this is the characteristic of the patient presented here). This toxicity can be induced by penicillin, estrogens, oral contraceptives, macrolides, and so forth, [6–12]. Direct toxicity is a dose-dependant ADR; its incidence is proportional to the dose of the drug, and when the drug is withdrawn, the pathologic findings are usually resolved, except for the serious cases with severe liver failure that may need liver transplant. Examples of drugs that have frequently induced direct toxicity are methotrexate, statins, and acetaminophen. Some chemotherapeutic drugs can alter specifically some transporting pumps and increase mainly total bilirubin without altering other biochemical tests. This represents a form of dose-dependent direct toxicity. Adalimumab-related DILI seems to be idiosyncratic, dose independent, and caused by an immune-mediated response with infiltration of the liver by eosinophils. The response to drug withdrawal is uncertain and the exact evolution is only known with a strict followup of the patient. Rechallenge is not recommended since a recurrent injury may be more severe than the first insult. Adalimumab-induced liver injury is a very rare ADR. Most of the patients with liver function test alterations during adalimumab treatment usually experience a reactivation of occult viral hepatitis (mainly hepatitis B virus) [13–20] or DILI because of other drugs such as MTX that are with some doubts linked to liver toxicity. In the case that we present, liver toxicity was temporally associated with symptoms, and liver function tests alterations improved when adalimumab was withdrawn. The pattern was cholestatic as few other case reports of adalimumab-induced hepatotoxicity [21, 22]. This patient can present doubts about the origin of the biochemical alterations either because of HCV infection or adalimumab toxicity. Generally the changes in the liver function tests due to HCV virus show a predominant rise in transaminases, and it is not very common for a cholestatic pattern to appear. Besides, it is not common at all to find portal infiltration with eosinophils in the liver biopsy of an HCV patient. To assess the likelihood of a causal association between adalimumab exposure and the liver toxicity, the drug-induced liver injury diagnostic scales were used. The degree of association between the implicated drug and the cholestatic damage was graded as “highly probable” using the Roussel Uclaf Causality Assessment Method (RUCAM) scale [23, 24] (score of 10) and “probable” using the Maria and Victorino [24] (score of 14) scales. 

Those are the reasons why we think that adalimumab was responsible for the liver toxicity in this patient. 

## 4. Conclusion

 In the case reported, adalimumab appears to be responsible for the liver injury because of the temporal relationship between the administration of the drug and the onset of pruritus and liver function tests alterations, the liver biopsy findings, other clinical conditions being discarded, and the improvement of clinical symptoms and biochemical abnormalities when adalimumab was suspended.
